# 原发性皮肤CD8阳性侵袭性嗜表皮细胞毒性T细胞淋巴瘤1例

**DOI:** 10.3760/cma.j.cn121090-20241125-00478

**Published:** 2025-04

**Authors:** 开婷 龙, 珍红 濮, 文秀 杨

**Affiliations:** 贵州医科大学，贵阳 550004 Guizhou Medical University, Guiyang 550004, China

患者，女，32岁，因“全身丘疹3个月”入院，患者颈部、肩背部、前胸部、双臂、臀部、腹股沟区、外阴、双大腿等部位无明显诱因出现绿豆至鸽蛋大小散在或密集分布的孤立不融合红色丘疹、斑块、水疱、暗红色色素沉着斑。浅表B超提示双侧腹股沟区和腋窝未探及明显异常结构淋巴结。胸部及全腹CT平扫检查提示：①左肺下叶结节；②副脾；③腹壁皮肤多发结节状增厚。实验室检查：尿液检查、大便检查、C反应蛋白、ANA抗体谱及凝血全套结果正常。血常规：WBC 11.06×10^9^/L、淋巴细胞百分比16.1％、中性粒细胞绝对值8.13×10^9^/L。肿瘤全套：鳞状细胞抗原（SCC）4.3 ng/ml（正常范围：0～2.50 ng/ml）。红细胞沉降率：39 mm/1 h（正常范围：0～26 mm/1 h）。病毒抗体全套：Ⅰ、Ⅱ型单纯疱疹病毒免疫球蛋白（Ig）G及人类巨细胞病毒IgG阳性。EB病毒感染相关抗体检测：EB病毒核抗原抗体IgG类阳性、EB病毒衣壳抗原抗体及EB病毒衣壳抗原低亲和力抗体IgG类可疑阳性。患者在外院诊断考虑鳞状细胞癌，为明确诊断，遂入我院皮肤科进行手术取右腰背部溃疡、左臂水泡、腹壁增殖处皮肤及穿刺骨髓组织送检。

病理检查肉眼观：右腰背部溃疡：皮肤及皮下组织一块，大小1.0 cm×1.0 cm×0.2 cm，皮肤表面破溃，未见肿块；左臂水泡：皮肤及皮下组织一块，大小1.0 cm×0.7 cm×0.2 cm，皮肤中央见一灰褐色隆起，高出皮表0.1 cm；腹壁增殖处：皮肤及皮下组织一块，大小1.3 cm×0.8 cm×0.4 cm，皮肤中央见一灰褐色隆起，高出皮表0.2 cm，表面欠光滑，切面灰白、实性、质中。镜检：右腰背部溃疡：皮肤及皮下组织，皮肤慢性溃疡形成，表皮轻度增生，表皮层见炎症细胞浸润及小脓肿形成，中等大小异型淋巴样细胞具有亲表皮的特点，细胞核呈多形性，可见散在母细胞样的核，细胞的浸润方式似乳腺派杰样方式，真皮内见大量中等大小异型淋巴样细胞、浆细胞、嗜酸性粒细胞浸润及组织细胞反应；左臂水泡：皮肤及皮下组织，表皮棘层松解，见表皮内水泡形成，真皮内见大量中等大小淋巴细胞、浆细胞、嗜酸性粒细胞浸润及组织细胞反应；腹壁增殖处：皮肤及皮下组织，皮肤慢性溃疡形成，真皮内纤维组织增生，并见大量中等淋巴样细胞、浆细胞、嗜酸性粒细胞浸润及组织细胞反应。免疫表型：LCA（+）、CD7（+）、CD3（部分+）、CD8（大部分+）、βF1（+）、CD4（少数+）、CD2（−）、CD5（−）、TIA1（+）、颗粒酶B（少数+）、CD43（弱+）、CD10（少数+）、CD68（−）、CD57（−）、CD34（−）、CD56（−）、S100（−），CD20（−）、CD30（−），TdT（−）、Ki-67增殖指数约70％。免疫组化染色结果提示皮肤病变增生的淋巴细胞中T细胞增生占明显优势、CD8阳性细胞明显多于CD4阳性细胞、CD2和CD5表达缺失、细胞Ki-67增殖指数高。EB病毒原位杂交阴性。T/B淋巴细胞基因重排检测：T淋巴细胞：TCRB A管（Vβ+Jβ1/2）、TCRB B管（Vβ+Jβ2）、TCRB C管（Dβ+Jβ1/2）及TCRG A管（Vγ1-8，Vγ10）阳性；B淋巴细胞：检测阴性。骨髓穿刺：骨髓造血功能增生活跃，三系未见明显异常，未见淋巴瘤累及。

病理诊断：结合临床、组织学、免疫表型及基因检测结果，皮肤病变为外周成熟的T细胞非霍奇金淋巴瘤，侵袭性，符合原发性皮肤CD8阳性侵袭性嗜表皮细胞毒性T细胞淋巴瘤。

患者经5个周期的西达本胺+CHOEP（环磷酰胺+脂质体阿霉素+长春地辛+依托泊苷+地塞米松）方案治疗，皮疹较前消退，渗出较前减少，部分皮疹结痂。患者化疗期间反复发热伴恶心、呕吐，完善皮肤分泌物细菌培养及鉴定：金黄色葡萄球菌。予以万古霉素抗感染后体温控制。排除造血干细胞采集禁忌证后行自体造血干细胞移植，移植方案为PD1+BEAC（卡瑞利珠单抗+苯达莫司汀+依托泊苷+阿糖胞苷+环磷酰胺）方案；在移植治疗期间，患者出现腹痛伴绞痛，完善淀粉酶未见明显异常，予以山莨菪碱解痉并加强护胃治疗后好转，出院后患者放弃治疗，确诊7个月后死亡。

